# How to evaluate a sexual health education intervention: experience from an Italian pilot project

**DOI:** 10.1093/eurpub/ckac129.743

**Published:** 2022-10-25

**Authors:** M Ubbiali, L Mortari

**Affiliations:** Università di Verona, Verona, Italy

## Abstract

**Introduction:**

Evaluation is an essential dimension of every educational activity, however it is a very crucial and problematic aspect to be considered. It is necessary to focus on some fundamental concepts: the idea of ‘measurement’, the definition of expected goals for the educational activity, whether they are measurable, and if not, how they can be evaluated. Finally, effectiveness may be assessed only after longer periods of time and by adopting coherent instruments. Outcomes of educational activities on sensitive themes such as sexuality education, are challenging to evaluate through causal relationships. So a fundamental question is: what is really possible to evaluate in the field of sexuality education (SE)?

**Methods:**

A desk review was carried out to collect information about national policies, international literature and guidelines on SE evaluation. A literature review was performed to collect and collate reported field experience and evaluation data.

**Results and discussion:**

In literature it is possible to find a consistent number of studies aimed at evaluating SE programs (in particular inspired by the models ‘abstinence-only’ and ‘abstinence-plus'), whose goal is to understand their impact on adolescents sexual health. Most of the studies reported limited evidences on SE efficacy on sexual health-related outcomes. This may be attributed to two different causes: methodological - evaluation instruments are epistemologically difficult to develop; and theoretical - the underlying philosophical frameworks they refer to may not fully reflect the complexity of sexuality education for adolescents.

**Conclusions:**

Based on the findings, evaluation instruments were developed to collect experiences from educators performing school-based sexuality education within the context of EduforIST project funded by the Italian Ministry of Health. Analysis of the collected data were still ongoing at the time of submission.

